# Role of the *Pseudomonas koreensis* BB2.A.1 and *Serratia liquefaciens* BB2.1.1 Bacterial Strains in Maize Trace Metal Stress Management

**DOI:** 10.3390/microorganisms12091823

**Published:** 2024-09-03

**Authors:** Éva-Boglárka Vincze, Annamária Becze, Rozália Veronika Salamon, Szabolcs Lányi, Gyöngyvér Mara

**Affiliations:** 1 Faculty of Science, Doctoral School of Chemistry, University of Pécs, Vasvári Pál Street 4., 7622 Pécs, Hungary; vinczeboglarka@uni.sapientia.ro; 2 Faculty of Economics, Socio-Human Sciences and Engineering, Sapientia Hungarian University of Transylvania, Libertăţii sq., 1, 530104 Miercurea Ciuc, Romania; beczeannamaria@uni.sapientia.ro (A.B.); salamonrozalia@uni.sapientia.ro (R.V.S.); lanyiszabolcs@uni.sapientia.ro (S.L.); 3 Faculty of Applied Chemistry and Material Sciences, Department of Analytical Chemistry and Environmental Engineering, Politehnica University of Bucharest, 1-7 Polizu Street, 011061 Bucharest, Romania

**Keywords:** PGPR, maize, plant growth, trace metal, abiotic stress, accumulation

## Abstract

Plant-growth-promoting rhizobacteria (PGPR), in addition to their well-known direct effects on plant growth and development, have been reported to be effective in plant abiotic (trace metal, drought, etc.) and biotic (phytopathogens, insects, etc.) stress management. PGPRs are involved in shaping the fate of trace metals in the rhizosphere and plants and thus may also reduce trace metal stress in plants. The aims of our study were to isolate and select indigenous trace-metal-resistant PGP strains and investigate their effects on maize germination and early development. The roles of the two selected strains, *Pseudomonas koreensis* and *Serratia liquefaciens* isolated from trace-metal-contaminated soil were investigated to mitigate trace metal stress in 21-day-old *Zea mays* seedlings. In the present study, 13 bacterial strains were isolated and screened for PGP traits under normal and trace metal stress conditions. The effect of two selected strains was further studied on plant experiments. The germination process, plant growth parameters (length, weight, dry matter content), photosynthetic activity, GPOX activity, trace metal accumulation, and translocation in microbes inoculated Cd (0.5 mM), Zn (1 mM), and Cd + Zn (0.1 + 0.5 mM) treated maize plants was studied. Our results revealed that trace metal toxicity, in terms germination and growth parameters and antioxidant enzyme activity, was enhanced upon inoculation with *Pseudomonas koreensis* BB2.A.1. Chlorophyll content and accumulation studies showed enhanced results following inoculation with *Serratia liquefaciens* BB2.1.1. Therefore, both bacterial strains possessed beneficial traits that enabled them to reduce metal toxicity in maize.

## 1. Introduction

Crop plants, such as rice, maize, wheat, soybean, and sorghum, are widely cultivated for food and feed in large quantities. The excessive use of agricultural land affects crop yield, and a wide variety of biotic and abiotic stresses can decrease the growth of these plants. The most important challenge in agriculture is to increase the nutrient use efficiency (NUE) and crop productivity through better crop management [1]. To achieve a high yield of crop plants in agricultural practice, a large variety of chemical fertilizers are used, with serious consequences, such as deterioration of the soil and environmental quality, affecting the health of living organisms. The use of biofertilizers based on plant-growth-promoting rhizobacteria (PGPR) is an alternative method for sustainable agriculture [2]. These bacterial strains can stimulate the growth and productivity of plants by improving the absorption and assimilation efficiency of nutrients and abiotic stress tolerance. Generally, biostimulants affect primary metabolism by increasing photosynthetic activity and generating compounds and can also induce secondary metabolism by activating specific biosynthetic pathways [3].

After molecular communication, PGPR colonizes the roots and rhizosphere of plants and can increase plant growth, development, and nutrient use efficiency. However, chemical fertilizers decrease their number and persistence [1,4]. The symbiotic and free-living beneficial microorganisms belong to the genera *Rhizobiaceae*, *Pseudomonas*, *Bacillus*, *Azotobacter*, *Arthrobacter*, *Serratia*, *Variovorax*, and *Azospirillum* [1,5].

PGPR help adjust the growth, development, and defense of plants by promoting plant growth through a variety of mechanisms, including biological nitrogen fixation, 1-aminocyclopropane-1-carboxylate (ACC) deaminase activity, phosphate solubilization, siderophore production, and hormonal compounds (indole-3-acetic acid, ethylene, gibberellin, and cytokinin) [6,7].

Plants need several nutrients for their normal growth and development, such as macro- (Ca, Mg, O, C, H, S, N, P, K) and micronutrients (B, Cu, Mn, Fe, Mo, Zn, Ni, Co, Cl) [7]. In addition, other metals are naturally present in the soil as compounds or simple ions. Trace metals are elements derived from parent rock and are found in the Earth’s crust; they have metallic characteristics such as conductivity, stability, and ligand specificity [8]. Anthropogenic activities, such as manufacturing and mining metals, pesticide application, and irrigation with untreated sewage water and sludge can cause environmental problems in the soil [9,10]. The most commonly used trace metals are As, Cr, Zn, Cu, Cd, Hg, Al, Be, and Pb [10].

Cd is considered the most phytotoxic trace element because it is highly soluble in water; therefore, it is easily taken up by plants. Thus, Cd enters the food chain and causes serious human health hazards. Cadmium generally occurs in the lithosphere (0.2 mg/kg), sedimentary rocks (0.3 mg/kg), and the soil (0.53 mg/kg). The use of phosphate fertilizers is one of the major anthropogenic Cd sources in agricultural soils, and the concentration of Cd in phosphate fertilizers can reach the range of 0.1–120 mg Cd (kg P_2_O_5_)^−1^. The highest cadmium concentrations in soil were reported in France (16.7 mg/kg), Belgium (7.61 mg/kg), and China (7.43 mg/kg) [11]. Sewage irrigation has contributed to Cd pollution in China over the past six decades, with more than 125.893 tons of Cd released into the environment [12].

The toxic effects of Cd on most plants range between 5 and 10 μg Cd/g dry matter [11]. Even at low concentrations, cadmium can severely alter several enzyme activities involved in the Calvin cycle, carbohydrate and phosphorus metabolism, and CO_2_ fixation. These effects result in stunted growth, chlorosis, leaf epinasty, alterations in chloroplast ultrastructure, inhibition of photosynthesis, pollen germination and tube growth, the induction of lipid peroxidation, the disruption of antioxidant machinery, and alterations in nitrogen and sulfur metabolism [13].

In contrast to Cd, the essential nutrient Zn plays an important role in the regulation of several physiological and biochemical processes in plants [14]. Cadmium and zinc have similar chemical and physical properties; therefore, these two metals compete for cell membrane transporters involved in uptake, translocation, sequestration, and remobilization [15]. The zinc concentration in soil varies between 10 and 300 mg/kg, and the average zinc content is approximately 50–55 mg/kg. Most plants contain high concentrations (30–100 mg/kg); however, concentrations higher than 300 mg/kg are generally toxic to plants [16]. On the other hand the majority of soils are zinc deficient; for example, in Indian soils, 0.01–52.9 mg/kg zinc is available, which is less than 1% of total required zinc content for plants [17]. Therefore, high concentrations of toxic trace metals, especially cadmium in soil, or a deficit of essential micronutrients such as zinc, can be an abiotic stress factor for plants.

PGPRs have mostly been used to assist plants in the uptake of large amounts of nutrients from the soil environment, as well as in the bioremediation of organic metal pollutants. Bacteria play a role in the immobilization of trace elements and limit their translocation to plants through precipitation, complex formation, and adsorption.

The aims of this study were multiple: (i) the isolation of indigenous trace-metal-tolerant bacterial strains; (ii) the assessment of the PGP activity of isolated bacterial strains under stress conditions; (iii) the inoculation effect of trace-metal-tolerant PGP bacteria on seed germination, plant growth parameters (length, weight, and dry matter content), physiology (photosynthetic activity), stress management (GPOX activity), and the accumulation and translocation of Cd and Zn in trace-metal-treated maize; (iv) the evaluation of trace metal uptake in the microbially inoculated maize.

## 2. Materials and Methods

### 2.1. Isolation of Trace Metal Resistant Bacterial Strains

Microbial isolates were recovered from a trace-metal-polluted soil from a spoil tip (Bălan area, Romania) using the serial dilution procedure. The isolation of soil bacteria was accomplished using agar media containing trace metals (0.5 mM Cd^2+^ + 1 mM Zn^2+^). One mL of soil suspension from dilutions 10^−1^–10^−10^ was added to sterile Petri plates and incubated at 28 °C for 72–120 h. Following the incubation, distinct colonies were picked up and transferred to Nutrient agar media and stored at 4 °C in a refrigerator for later use. A total of 13 isolates were obtained.

### 2.2. Assessment of PGP Traits under Trace Metal Stress

The mobilization ability of organic and inorganic phosphorus and the production of siderophores, indole acetic-3-acid (IAA), and exopolysaccharide (EPS) of trace-metal-tolerant bacterial strains were determined. These properties are the basis for the selection of bacterial strains for plant experiments.

#### 2.2.1. Determination of Siderophore Production

The ability of the bacterial strains to produce siderophores was examined on solid culture media containing chrome azurol S (CAS), as described by Oldal et al. (2002), amended with trace metals [18]. Bacterial suspensions (OD_590_ = 0.3) were obtained in physiological solution from cultures grown overnight. The media were inoculated with 10 µL of the bacterial suspension using spot inoculation. Samples were prepared in triplicate and incubated at 28 °C for 72 h. The plates were examined for the development of a yellow zone (halo) around the colonies, indicating the release of siderophores. The diameter of the halo zone was measured using digital calipers.

#### 2.2.2. Mobilization of Organic and Inorganic Phosphorus

Organic phosphorus mobilization ability was tested on solid culture media containing Na-phytate (glucose 15 g/L, (NH_4_)_2_SO_4_ 5 g/L, KCl 0.5 g/L, MgSO_4_·7H_2_O 0.1 g/L, NaCl 0.1 g/L, CaCl_2_·2H_2_O 0.1 g/L, FeSO_4_·7H_2_O 0.01 g/L, MnSO_4_ 0.01 g/L, Na-phytate 5 g/L, agar 20 g/L) amended with trace metals. The medium was inoculated with 10 µL of the bacterial suspension (OD_590_ = 0.3) using spot inoculation and incubated at 28 °C for 72 h. The plates were examined for the development of a clear zone (halo) around the colonies, indicating the production of phytase. The diameter of the halo zone was measured using digital calipers. The samples were prepared in triplicate.

Inorganic phosphorus mobilization was determined using spot inoculation on Pikowskaya solid medium (yeast extract 0.5 g/L, glucose 10 g/L, calcium phosphate 5 g/L, ammonium sulfate 0.5 g/L, KCl 0.2 g/L, MgSO_4_ 0.1 g/L, MnSO_4_ 0.0001 g/L, FeSO_4_ 0.0001 g/L, agar 20 g/L) amended with trace metals, as mentioned before [19].

#### 2.2.3. Evaluation of Indole-3-Acetic Acid Production

The indole-3-acetic acid (IAA) secretion by the isolated bacterial strains was measured using the methodology described by Becze et al. (2021) [20]. Bacterial strains were grown in 5 mL TSB (tryptone–soy broth) amended with trace metals and incubated at 28 °C and 150 rpm for 72 h in a shaking incubator. After incubation, 1.5 mL of the bacterial cultures was centrifuged (5.000 rpm, 15 min) and 1 mL of the supernatant was mixed with 2 mL of Salkowsky reagent (300 mL of 98% H_2_SO_4_, 15 mL of 0.5 M FeCl_3_, and 500 mL of distilled water). For color development, the prepared samples were incubated at room temperature in the dark for 30 min. The absorbance of the samples was measured at 570 nm by measuring the pink color that emerged during the reaction. The indole-3-acetic acid concentration was determined using IAA as a reference (calibration curve).

#### 2.2.4. Swimming and Swarming Motility

The swimming and swarming motility of the isolated bacterial strains was determined using Luria Bertani (LB) agar media (NaCl 10 g/L, peptone 10 g/L, yeast extract 5 g/L, pH = 7). For the swarming test, the medium was supplemented with 0.5% agar, and for the swimming test, it was supplemented with 0.3% agar [21]. The bacterial strains were grown overnight on Nutrient agar media, and the Petri plates were inoculated in triplicates. After five days of incubation at 28 °C, the diameters of swimming and swarming zones were measured, and the results were compared.

### 2.3. Seed Germination Experiments

The selected bacterial strains were used in the maize germination experiments. To sterilize maize seeds, they were first treated with absolute ethanol for one minute, followed by three washes with distilled water. The seeds were then treated with a hypochlorite solution for 20 min and washed three times with distilled water. The surface-sterilized seeds were soaked for 30 min in bacterial suspension (OD_590_ = 1) for bacterial treatment and in nutrient broth for the control. Germination was carried out in Petri dishes (10 seeds each, 8 replicates) in the dark at 28 °C for eight days, and the seeds were sprinkled twice with distilled water (2 mL) on days four and six. The number of germinated seeds was counted every two days during the experiment. At the end of the germination experiment, the following germination indices were calculated: germination rate (G), mean germination time (MGT), coefficient of velocity of germination (CVG), mean germination rate (MGR), germination rate index (GRI), germination index (GI), and Timson Germination Index (TGI) [22].

**G** = (total seeds germinated at the end of trial/total number of initial seeds) × 100.

MGT=∑Fx/∑F

where F is the number of seeds germinated on day x.

CVG=(N1+N2+…+Ni)/100×(N1T1+…+NiTi)

where N is the number of seeds germinated every day and T is the number of days from seeding corresponding to N.

MGR=CVG/100=1/T

where T is the mean germination time and CVG is the coefficient of velocity.

GRI=G1/1+G2/2+….+Gi/i

where G1 is the germination percentage on day 1, G2 is the germination percentage on day 2; and so on.

GI=(8×N1)+(6×N2)+…+(2×N8)

where N1, N2, …, N8 are the number of germinated seeds on the first, second and subsequent days until 8th day, and the multipliers (for example 8 and 6) are the weights assigned to the days of germination.

**TGI** = ΣG/T

where G is the percentage of seeds germinated per day and T is the germination period.

### 2.4. Identification of Bacterial Isolates

Two bacterial strains were selected from the 13 trace-metal-tolerant bacterial strains, which were marked as BB2.A.1 and BB2.1.1. Pure bacterial cultures were obtained on Nutrient agar plates by the streak plate method. Bacterial cells were stained with methylene blue (1%) and observed under an optical microscope (Olympus BX53, Olympus Corporation, Tokyo, Japan). Pictures were obtained using labSens 1.1. software. These bacterial strains were identified by 16S rDNA sequence analysis. The sequences were edited and aligned using Chromas 2.6.6. software. The sequences were compared with those found in the NCBI database and deposited in the EMBL database. The phylogenetic tree was constructed using MEGA 11.0 software.

### 2.5. Effect of PGP Bacterial Strains in Plant Growth Experiments

The effects of PGP bacterial strains were tested on maize (*Zea mays* MV3122 certified variety) plants under stress conditions. Maize seeds were surface sterilized with absolute ethanol for 1 min and then washed with distilled water three times. The seeds were then soaked in a hypochlorite solution for 20 min and rinsed several times with distilled water. Germination was performed in the dark on wet filter paper in Petri dishes at 28 °C for five days, and water was replenished once with 2 mL of distilled water. In the next phase, the sterilized soil (121 °C, 40 min) was placed in sterile polypropylene boxes. Twenty equally developed plant seeds were planted in each pot, and the bacterial suspension (0.5 mL/seed, OD_590_ = 1.5) was pipetted into the seed environment. After that, the soil was treated with different concentrations of trace metal (0.5 mM Cd^2+^, 1 mM Zn^2+^, 0.1 mM Cd^2+^ + 0.5 mM Zn^2+^). Finally, the seedlings were placed in a plant growth chamber (Sanyo MLR-351 Versatile Environmental Test Chamber, SANYO Electric Co., Ltd., Osaka, Japan) for 21 days under controlled conditions: 22 °C, 70% relative humidity, and 12 h/day 2500 lx light.

### 2.6. Plant Growth Parameters

Plant growth parameters were measured at the end of the experiment. The weights of the shoots and roots were determined using a digital scale (KERN 440-33, KERN & Sohn GmbH, Balingen, Germany), and the length was measured using a digital caliper. The numbers of leaves and root hairs were also counted.

### 2.7. Chlorophyll Content of the Maize Plants

The extraction of total chlorophyll (chlorophyll-α and chlorophyll-β) was carried out using an ammonia–acetone solution (1 L of 80% acetone, 1.5 mL of 25% ammonia solution). First, the plant leaf samples (0.15 g) were destroyed in a mortar and pestle with an ammonia acetone solution. The plant extracts were centrifuged at 5000 rpm for 5 min in centrifuge tubes, and the supernatants were transferred into new tubes and filled up to 5 mL with the ammonia–acetone solution. The absorbance of the prepared samples was measured with a spectrophotometer (HACH DR 6000, Hach Company, Loveland, Colorado, USA) at 800, 730, 664, and 647 nm. Measurements at 800 nm and 730 nm are required as correction factors for acetone absorption; 664 nm is chlorophyll-α, and 647 nm is the chlorophyll-β absorption maximum. The amounts of chlorophyll-α and chlorophyll-β were calculated from the absorbance values based on the method described by Porra et al. [23].

### 2.8. Determination of GPOX Enzyme Activity

During sample preparation, 0.15 g of plant shoots and roots were weighed, which was first disrupted in a mortar with a pestle, and then treated with 1 mL of QB buffer (pH 7.8, 100 mM KPO_4_, 1 mM EDTA, 1% Triton X-100, 10% glycerol, 1 mM dithiothreitol, DTT added only before use) and centrifuged at 14,000 rpm for 30 min at 4 °C, and the supernatant was stored at −20 °C.

The total protein concentration was determined using the Bradford method [20]. Guaiacol peroxidase (GPOX) enzyme activity was measured using a spectrophotometric method. The reaction mix contained the following solutions: 885 µL 1 M Na-phosphate buffer, 50 µL 50 mM guaiacol solution, 50 µL H_2_O_2_, and 15 µL extracted plant sample. The absorbance was measured (Agilent Technologies Cary 60 UV-VIS, Agilent, Santa Clara, CA, USA) at 436 nm after 5 s for 15 min [24].

### 2.9. Determination of Trace Metal Accumulation in Plants

To determine the accumulation of trace metals, samples were dried to a constant weight at 105 °C in a heat sterilizer. The pre-dried samples were placed in a porcelain jar and incinerated in a Gefran 1001 incinerator at 450 °C for 4 h, with a continuous increase in temperature from 250 °C to 100 °C per hour. The resulting ash was dissolved in 5 mL of 25% HNO_3_, filtered through a filter paper, and filled with distilled water to a volume of 10 mL [20]. The trace metal content of the solution was determined using a Varian Spectra AA 110 atomic absorption spectrophotometer. Concentrations were determined using a calibration curve obtained using standard solutions [20]. To compare the root-to-shoot translocation of Zn and Cd between the different treatments, the translocation factor (TF) was determined as the ratio of shoot/root Zn and Cd concentrations.

### 2.10. Evaluation of Inoculum Persistence

The number of bacterial cells was determined by re-isolation. The importance of re-isolation was to detect the presence of bacteria after plant experiments and to compare the changes in the number of bacterial cells. The number of colonies was determined using the spread plate method for colony counting.

### 2.11. Statistical Analysis

Data presentation and statistical analysis were carried out using Microsoft Excel 2019 and the Past.exe 4.03 statistical program. One-way ANOVA was used to compare the data series between treatments and controls, followed by Tukey’s test and Pearson correlation analysis.

## 3. Results

### 3.1. Bacterial Strains

In this study, 13 bacterial strains from trace-metal-polluted soil from a spoil tip (Bălan area, Transylvania, Romania) were isolated, and 11 of the isolates were assessed for their beneficial traits. Five strains proved to be strong siderophore producers, seven strains were able to mobilize inorganic phosphorus, and four strains produced higher amounts of IAA. All strains produced EPS and showed both types of motilities (Table 1). Each beneficial trait was converted to a value from 0 to 5, the values were added together and compared. From the 11 isolated bacterial strains, two strains (BB2A1 and BB211) with the highest values based on all beneficial traits were selected for further analysis.

**Figure 1:**
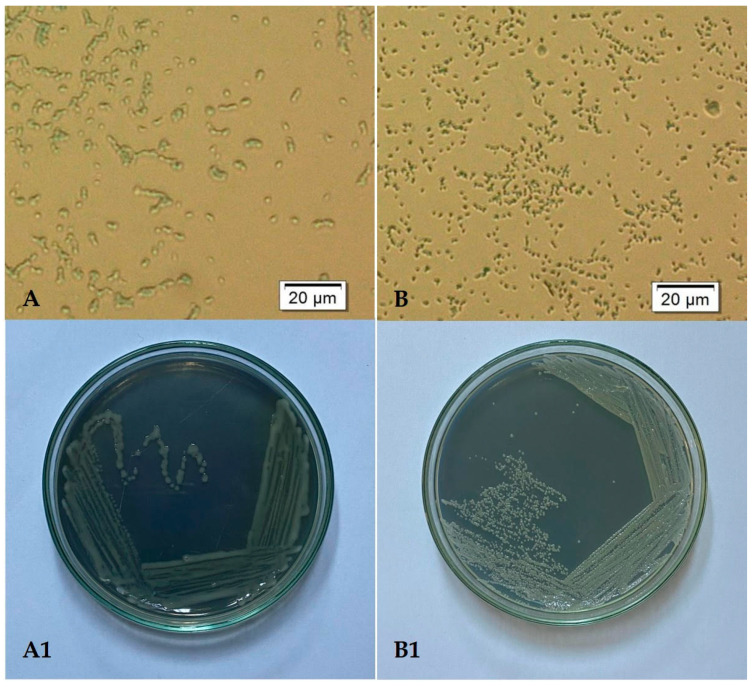
Microscopic picture of *Pseudomonas koreensis* BB2.A.1 at 400× magnification (**A**) and its culture on Nutrient agar plate (**A1**). Microscopic picture of *Serrratia liquefaciens* BB2.1.1 at 400× magnification (**B**) and its culture on Nutrient agar plate (**B1**).

**Figure 2:**
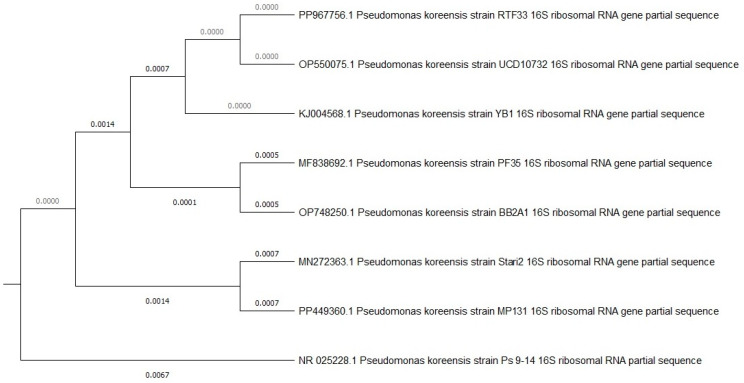
Phylogenetic tree based on a comparison of the 16S ribosomal DNA partial sequences *Pseudomonas koreensis* BB2.A.1 strain and some closest phylogenetic relatives. The tree was created using the neighbor-joining method.

**Figure 3:**
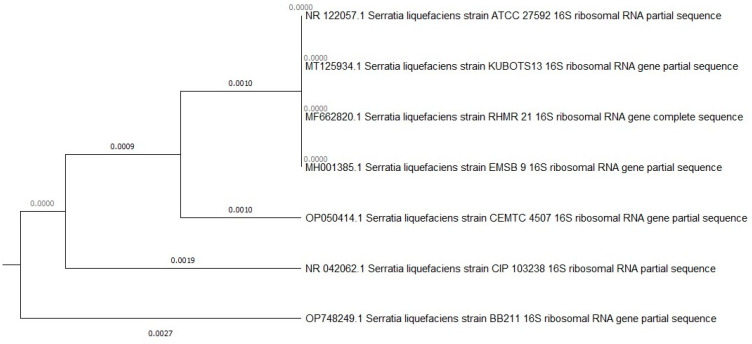
Phylogenetic tree based on a comparison of the 16S ribosomal DNA partial sequences *Serratia liquefaciens* BB2.1.1 strain and some closest phylogenetic relatives. The tree was created by the neighbor-joining method.

Both selected bacterial strains were identified using 16S rDNA sequence analysis performed by BIOMI Ltd. (Godollo, Hungary). GenBank accession numbers are given in parentheses after the species names. The two bacterial strains identified were *Pseudomonas koreensis* BB2.A.1 (OP748250) and *Serrratia liquefaciens* BB2.1.1 (OP748249). The cell and colony morphologies of both the strains are presented in Figure 1. The phylogenetic trees of the two bacterial strains are presented in Figure 2 and Figure 3.

### 3.2. Plant Growth Promoting Potential of the Selected Strains under Trace Metal Stress

The siderophore production ability of both strains was modified in the presence of trace metals. The presence of Zn and lower Cd concentrations with Zn resulted in significantly higher values compared to the control (Table 2). The presence of 0.5 mM Cd lowered siderophore production in both strains. The phosphorus mobilization ability of the selected strains was also studied at different trace metal concentrations. Organic phosphorous mobilization ability was observed only in plates containing trace metals. Inorganic phosphorus mobilization in *P. koreensis* BB2.A.1 was lowered by the presence of 1 mM Zn and 0.5 mM Cd, whereas in the presence of both trace metals, the halo diameter was similar to that observed in the control samples. The inorganic phosphorus mobilization ability of the *Serratia liquefaciens* BB2.1.1. strain was not significantly affected by the presence of trace metals. Indole acetic acid production was slightly affected by the presence of both metals in both the bacterial strains. Exopolysaccharide production was completely inhibited by Cd in *P. koreensis* BB2.A.1, whereas the higher Cd concentration inhibited EPS production in *Serratia liquefaciens* BB2.1.1. too. Both the stimulatory and inhibitory effects of trace metal treatments on plant-growth-promoting characteristics were observed; however, total limitation was observed only in EPS production. Most of the PGP characteristics were retained despite the presence of Zn and/or Cd. Regarding bacterial trace metal stress reduction, the two important traits that may prevent trace metals from entering the plant cell, siderophore production and EPS production, were affected by metal concentration.

### 3.3. Germination of Bacteria-Treated Maize Seeds

Seed germination and seedling emergence are two of the most critical and delicate stages in the life cycle of a crop, as they lay the foundation for the plant’s future growth and development. Therefore, the selected bacterial strains were used to treat maize seeds. Table 3 shows the mean variations for the seven germination indices as affected by seed inoculation. Seeds treated with the *Pseudomonas koreensis* BB2A.1. strain increased by 2.47%, whereas inoculation with *Serratia liquefaciens* BB2.1.1 increased the seed germination rate by 12.47%. The average coefficient of velocity of germination (CVG) was 21.76 for control seeds and 24.43 (112.27%) and 29.99 (137.72%) for *P. koreensis* BB2A.1.- and *S. liquefaciens* BB2.1.1-treated seeds, respectively. The Timson Germination Index (TGI) was higher in inoculated seeds than in the control. The highest seed germination indices were achieved when inoculated with *S. liquefaciens* BB2.1.1, followed by that of *P. koreensis* BB2A.1. inoculation. However, the differences in the values were not statistically significant.

### 3.4. Growth and Development of the Maize Plants with PGPR Inoculation

#### 3.4.1. The Length of the Shoot and the Root of Maize Plants

**Figure 4:**
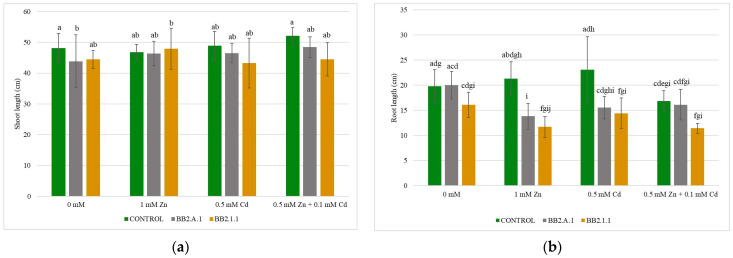
The length of the shoot (**a**) and the root (**b**) of the maize plants. Altogether 10 plants were selected randomly from each treatment. One-way ANOVA followed by a Tukey test was computed to compare variables (mean ± standard error). Different letters represent significant differences among the treatments according to Tukey’s test (*p* < 0.05).

Figure 4 and Table 4 shows the shoot and root lengths of maize plants. The *Pseudomonas koreensis* BB2.A.1 bacterial strain reduced the shoot and root length growth compared to the treated controls (1 mM Zn, 0.5 mM Cd, 0.5 mM Zn + 0.1 mM Cd). Samples treated and inoculated with the *Pseudomonas koreensis* BB2.A.1 bacterial strain were slightly longer than the inoculated control in the case of the shoot, but the root length decreased in the presence of used treatments.

The *Serratia liquefaciens* BB2.1.1 bacterial strain increased (by 6.66%) the shoot length of maize plants when 1 mM zinc concentration was applied, compared with the uninoculated and treated control. Nonetheless, in the presence of cadmium and combined treatment, the *S. liquefaciens* BB2.1.1 bacterial strain decreased shoot length (by 11.51%, 14.63%). However, these differences were not statistically significant. A decreasing tendency can be observed in the case of root length, because the *S. liquefaciens* BB2.1.1 bacterial strain and the concentrations used reduced the examined parameter (by 10.37%–45.12%) compared to the inoculated and uninoculated controls.

#### 3.4.2. The Weight of the Shoot and the Root of the Maize Plants

**Figure 5:**
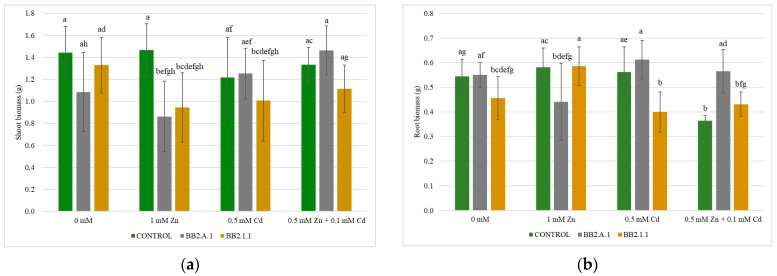
The weight of the shoot (**a**) and the root (**b**) of the maize plants. Altogether 10 plants were selected randomly from each treatment. One-way ANOVA followed by a Tukey test were computed to compare variables (mean ± standard error). Different letters represent significant differences among the treatments according to Tukey’s test (*p* < 0.05).

The shoot and root biomass results are shown (Figure 5 and Table 5). The *Pseudomonas koreensis* BB2.A.1 bacterial strain had a similar effect on the shoot and root biomass, and the bacterial inoculation decreased the biomass in the presence of 1 mM Zn treatment and increased it in the case of the 0.5 mM Cd and combined treatment (0.5 mM Zn + 0.1 mM Cd). These differences were observed compared to the uninoculated and treated samples and relative to the inoculated control.

The *Serratia liquefaciens* BB2.1.1 bacterial strain decreased the shoot biomass in all treatments compared to the uninoculated and treated samples, whereas the root biomass decreased only in the presence of 0.5 mM Cd treatment. The combined treatment increased the root biomass, whereas the zinc treatment had no effect on this parameter. The treatments had a different effect on the root biomass compared to the inoculated control, because the zinc concentration increased (22.41%), while the Cd (11.11%) and combined treatments (4.44%) slightly decreased the biomass values.

#### 3.4.3. The Dry Matter Content (DMC) of the Shoot and the Root of the Maize Plants

**Figure 6:**
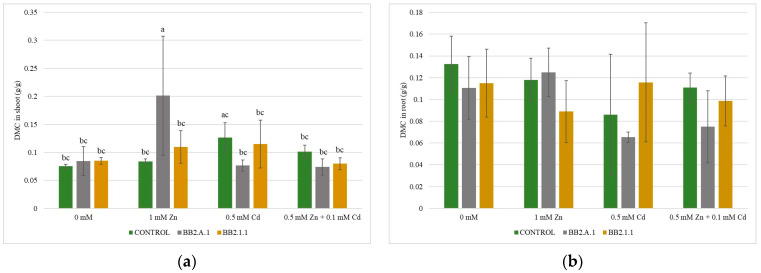
The DMC values of the shoot (**a**) and the root (**b**) of the maize plants. Altogether, 5 plants were selected randomly from each treatment. One-way ANOVA followed by a Tukey test was computed to compare variables (mean ± standard error). Different letters represent significant differences among the treatments according to Tukey’s test (*p* < 0.05).

The DMC results are shown in Figure 6. Zinc treatment (1 mM Zn) resulted in a remarkably high (50%) dry matter content (DMC) in the shoot of maize plants inoculated with *Pseudomonas koreensis* BB2.A.1 bacterial strain, compared to the uninoculated and treated control, as well as to the uninoculated and untreated controls. The differences were statistically significant. Inoculation with Cd (41.66%) or combined (30%) treatment resulted in a decreasing trend of DMC in the shoot, compared to the uninoculated and treated samples, as well as to the uninoculated and untreated control (12.5%). In the case of root DMC, the same results were observed: inoculation increased the DMC in the presence of zinc treatment (8.33%), whereas cadmium (25%, 45.45%) and combined treatment (36.36%) lowered DMC, compared to the uninoculated and treated samples, as well as to the uninoculated and untreated control.

When inoculated with *Serratia liquefaciens* BB2.1.1, the bacterial strain increased DMC in the shoot in the presence of zinc treatment (27.27%), whereas the cadmium (8.33%) and combined treatment (20%) resulted in lower values compared to the uninoculated and treated samples. In the presence of bacteria, the Zn and Cd treatments increased (27.27%), whereas the combined treatment did not influence the DMC values in the shoot compared to the uninoculated and untreated controls. In the case of the root DMC values, we observed that inoculation increased the parameter in the presence of zinc and combined treatment (18.18%), while the cadmium concentration decreased (27.27%) the DMC values compared to the uninoculated and treated samples. Regarding the uninoculated and untreated controls, the DMC in the roots varied as follows: inoculation decreased the DMC in the presence of zinc (18.18%) or combined treatment (9.09%).

### 3.5. Determination of Trace Metal Accumulation in Plants

**Figure 7:**
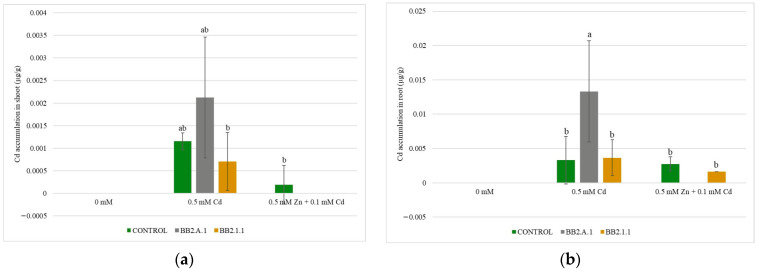
The Cd accumulation in the shoot (**a**) and the root (**b**) of the maize plants. Altogether, 5 plants were selected randomly from each treatment. One-way ANOVA followed by a Tukey test was computed to compare variables (mean ± standard error). Different letters represent significant differences among the treatments according to Tukey’s test (*p* < 0.05).

In the control and samples treated with both bacterial strains (lower Cd concentrations), the presence of Cd in plant tissues was not detectable. Although the measured values of Cd accumulation in the shoots and roots of maize plants in different treatments showed changes, these were not statistically significant (Figure 7). The presence of the *Pseudomonas koreensis* BB2.A.1 bacterial strain increased the accumulation of toxic metals in the shoot (47.61%) and the root (75.93%), in the case of the 0.5 mM Cd treatment, compared to the uninoculated and treated samples. The *Serratia liquefaciens* BB2.1.1 bacterial strain decreased (36.36%) the accumulation of Cd in the shoot in the presence of the 0.5 mM Cd treatment, compared to the uninoculated and treated samples. The presence of *S. liquefaciens* BB2.1.1 slightly increased (11.11%) the amount of accumulated cadmium in the roots in the case of the Cd treatment, whereas in the presence of the combined treatment, it decreased (40.74%) the accumulation of cadmium.

**Figure 8:**
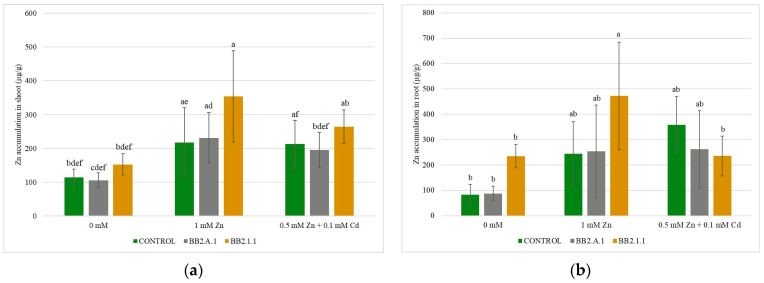
The Zn accumulation in the shoot (**a**) and the root (**b**) of the maize plants. Altogether, 5 plants were selected randomly from each treatment. One-way ANOVA followed by Tukey test was computed to compare variables (mean ± standard error). Different letters represent significant differences among the treatments according to Tukey’s test (*p* < 0.05).

The results of zinc accumulation measurements are shown in Figure 8. The *Pseudomonas koreensis* BB2.A.1 bacterial strain slightly increased the accumulation of zinc in the shoot and roots in the presence of Zn treatment, while in the case of the combined treatment, a slightly decreasing trend was observed compared to the uninoculated and treated samples. A statistically significant difference was observed only in Zn-treated samples (1 mM Zn), where the *Serratia liquefaciens* BB2.1.1 bacterial strain stimulated the accumulation of Zn in the shoot compared to the inoculated control (131.49%) samples. Inoculation with the *Serratia liquefaciens* BB2.1.1 bacterial strain and the Zn treatment significantly increased zinc accumulation in the roots compared to that in the inoculated control (101.56%).

A plant is efficient in metal translocation from the root to the shoot when the translocation factor (TF) is higher than one, owing to its efficient metal transport system. If the TF value is less than one, metal transfer is inhibited.

The translocation factor values for Cd were not obtained in the case of the control samples and combined treatments. The translocation values for control and inoculated samples treated with 0.5 mM Cd were low (0.08–0.16) because of the inhibited translocation from the root into the shoot.

The translocation of zinc as an essential micronutrient was determined, and the transport of accumulated zinc from root into the shoot was realized to a large extent (1.65 for the control, 1.14 for 1 mM Zn, and 0.64 for the combined treatment). The *P. koreensis* BB2.A.1 bacterial strain increased the TF of zinc in the case of 1 mM Zn (14.28%) and combined treatments (34.69%). The *S. liquefaciens* BB2.1.1 bacterial strain decreased the TF in the presence of 1 mM Zn treatment (46.49%), whereas the combined treatment increased the TF (47.10%). Both bacterial strains increased the TF of Zn in the combined treatment, stimulating the translocation of essential micronutrients to the shoot.

### 3.6. Chlorophyll Content of the Maize Plants

**Figure 9:**
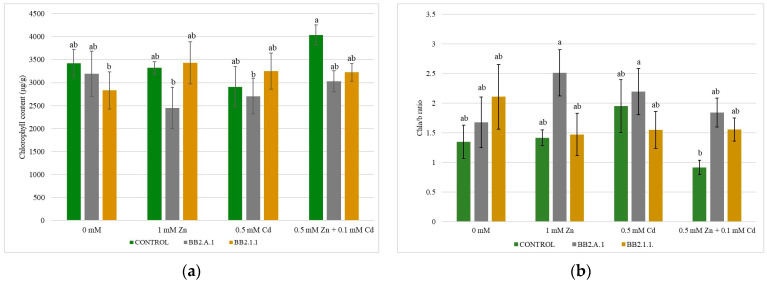
The total chlorophyll content (**a**) and the Chla/b ratio (**b**) of the maize plants. Altogether, 3 plants were selected randomly from each treatment. One-way ANOVA followed by a Tukey test was computed to compare variables (mean ± standard error). Different letters represent significant differences among the treatments according to Tukey’s test (*p* < 0.05).

No statistically significant differences were found in the total chlorophyll content of the maize plants (Figure 9). The *Pseudomonas koreensis* BB2.A.1 bacterial strain decreased (5.27–25%) the chlorophyll content of the maize plants in all the treatments, compared to the uninoculated and treated samples and also compared to the inoculated control. The *S. liquefaciens* BB2.1.1 bacterial strain slightly increased the chlorophyll content in the case of the 1 mM Zn (3.16%) and 0.5 mM Cd treatment (10.38%), whereas in the presence of the combined treatment, a decrease (20.02%) was observed. Inoculation with *S. liquefaciens* BB2.1.1 in all applied treatments, compared with the inoculated control, increased the chlorophyll content (26.23–30.61%).

The Chla/b ratio showed a reverse trend compared to the total chlorophyll. Both bacterial strains increased the Chla/b ratio of the control samples, whereas when the trace metal treatments were applied, the inoculation with *P. koreensis* BB2.A.1 bacterial strain increased the ratio in all treatments.

### 3.7. Determination of GPOX Enzyme Activity

**Figure 10:**
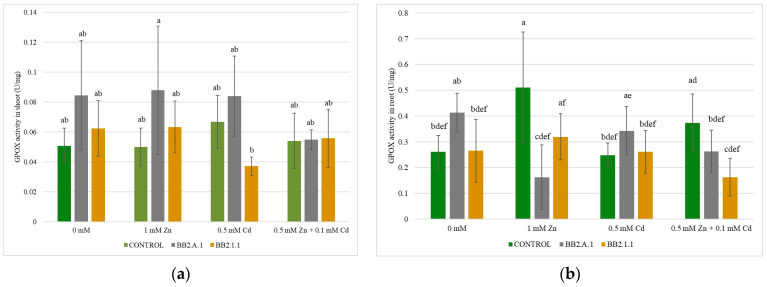
The GPOX activity in the shoot (**a**) and the root (**b**) of maize plants. Altogether, 5 plants were selected randomly from each treatment. One-way ANOVA followed by a Tukey test was computed to compare variables (mean ± standard error). Different letters represent significant differences among the treatments according to Tukey’s test (*p* < 0.05).

GPOX enzyme activity in the roots of maize plants was higher than that in the shoots. The differences in GPOX enzyme activity in the shoots of the maize plants were not statistically significant (Figure 10). The most significant effect on GPOX values in the shoots was caused by *Pseudomonas koreensis* BB2.A.1 bacterial strain, which increased the enzyme activity in the presence of 1 mM Zn (44.44%) and 0.5 mM Cd (12.50%) compared to the inoculated control. Inoculation with the above-mentioned bacterial strain in the case of the combined treatment had no significant effect on GPOX activity in the shoot compared to the inoculated control. The *Pseudomonas koreensis* BB2.A.1 inoculation lowered the root GPOX enzyme activity in the presence of the 1 mM Zn treatment and the combined treatment compared to uninoculated, treated samples (68.62% and 29.72%, respectively) in the inoculated control (60.77% and 36.58%, respectively). Inoculation and Cd treatment increased GPOX activity in the roots compared to that in the uninoculated and treated samples (29.41%).

The *Serratia liquefaciens* BB2.1.1 bacterial strain increased the GPOX activity in the shoot in the case of Zn (16.66%) and combined treatment (16.66%), whereas the Cd treatment (42.85%) decreased the activity compared to the uninoculated and treated samples. Bacterial inoculation decreased GPOX activity in the shoot in the presence of Cd (33.33%) compared to the inoculated control. The *Serratia liquefaciens* BB2.1.1 bacterial strain decreased GPOX activity in the roots in the presence of Zn (39.21%) and combined treatment (56.75%) compared to the uninoculated and treated samples. Regarding the inoculated control, the zinc treatment increased (16.12%), while the combined treatment (38.46%) decreased GPOX enzyme activity in the root.

### 3.8. Evaluation of Inoculum Persistence

Inoculum persistence was evaluated to monitor the change in the number of inoculated bacteria during the plant experiment. At the beginning of the experiment, the samples were inoculated with 816 × 10^6^ CFU/mL *Pseudomonas koreensis* BB2.A.1 bacterial strain and 231 × 10^6^ CFU/mL of *Serratia liquefaciens* BB2.1.1 bacterial strain.

Both the bacterial strains (*Pseudomonas koreensis* BB2.A.1 and *Serratia liquefaciens* BB2.1.1) were successfully isolated at the end of the plant experiments from control samples to the following extent: 968 × 10^6^ CFU/mL and 166.4 × 10^6^ CFU/mL. In the presence of 1 mM Zn, 0.5 mM Cd, and 0.5 mM Zn + 0.1 mM Cd treatments, the *Pseudomonas koreensis* BB2.A.1 bacterial strain was isolated at the following colony densities: 928 × 10^6^ CFU/mL, 536 × 10^6^ CFU/mL, and 1056 × 10^6^ CFU/mL. The *Serratia liquefaciens* BB2.1.1 bacterial strain was isolated with the following concentrations of trace metals: 1 mM Zn (312 × 10^6^ CFU/mL), 0.5 mM Cd (424 × 10^6^ CFU/mL), and 0.5 mM Zn + 0.1 mM Cd (1248 × 10^6^ CFU/mL). Two substantial differences in microbe number were observed: Cd treatment (0.5 mM Cd) decreased the quantity of the *Pseudomonas koreensis* BB2.A.1 bacterial strain at 34.31%, whereas the combined treatment increased the number of colony forming units of *Serratia liquefaciens* BB2.1.1.

### 3.9. Pearson Correlation Analysis

**Figure 11:**
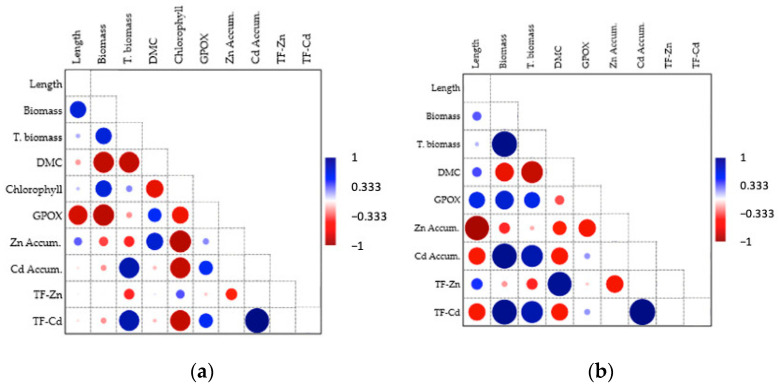
Pearson linear correlation between different shoot (**a**) and root (**b**) parameters in the case of the *P. koreensis* BB2.A.1 bacterial strain: length (cm), biomass (g), total biomass (g), dry matter content (DMC, g/g), chlorophyll (μg/g), guaiacol–peroxidase activity (GPOX, U/mg), Zn accumulation (μg/g), Cd accumulation (μg/g), Zn translocation factor (TF-Zn), Cd translocation factor (TF-Cd).

Figure 11 shows the Pearson correlation results for the shoot (a) and root (b) parameters in the context of *Pseudomonas koreensis* BB2.A.1 bacterial strain inoculation. The growth parameters (length, biomass, and total biomass) showed a positive correlation between them, because biomass changed in the same way with the length and, respectively, with the total biomass. The shoot biomass decreased compared to the DMC values; that is, the DMC and shoot biomass had a strong negative correlation. Similar results were observed for total biomass and DMC, which can be explained by the higher water content of the shoot.

Considering the growth and physiological parameters, a positive correlation was observed between biomass, DMC, and the chlorophyll content of the shoot. GPOX activity was strongly negatively correlated with the shoot growth parameters (length and biomass). This can be explained by the fact that if there is less stress in the shoot, growth is greater.

The growth parameters and metal accumulation in the majority were positively correlated. Zn accumulation was positively correlated with DMC values, whereas Cd accumulation showed a strong positive correlation with the total biomass of the plants. The two metal accumulation values were strongly negatively correlated with the chlorophyll content of the plants. In the presence of high metal concentrations, chlorophyll faded into the background.

In the case of the root, we observed a similar tendency between growth parameters (length, biomass, total biomass, and DMC) as in the case of the shoot, because DMC remained strongly negatively correlated with biomass. These results indicate that the difference in weight originates from the higher water content of the samples. The root relationships are the opposite of those experienced in the shoot.

Physiological and growth parameters were positively correlated. The GPOX activity values varied just as the growth parameters (length, biomass, and total biomass) did, indicating that the presence of the antioxidant system had a stimulating effect on plant growth parameters.

Considering the growth and accumulation parameter results, we observed the following correlations. A negative correlation was observed between Zn and Cd accumulation and the growth parameters (length and DMC). This phenomenon occurred because the accumulation of these metals in the shoot was lower than that in the roots. The relationship between root biomass and Cd accumulation in roots showed the same positive correlation as in the case of shoots. This correlation can be seen in the fact that Cd mostly accumulated in the roots, increasing the biomass. Zn accumulation was negatively correlated with GPOX activity. Zinc is an essential micronutrient that does not cause high stress in plants.

**Figure 12:**
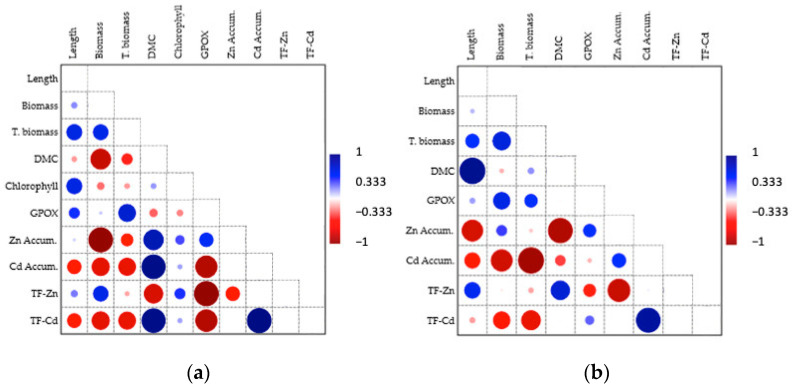
Pearson linear correlation between different shoot (**a**) and root (**b**) parameters in the case of the *S. liquefaciens* BB2.1.1 bacterial strain: length (cm), biomass (g), total biomass (g), dry matter content (DMC, g/g), chlorophyll (μg/g), guaiacol–peroxidase activity (GPOX, U/mg), Zn accumulation (μg/g), Cd accumulation (μg/g), Zn translocation factor (TF-Zn), Cd translocation factor (TF-Cd).

In Figure 12, Pearson correlation results can be observed for the shoot (a) and root (b) parameters based on *Serratia liquefaciens* BB2.1.1 bacterial strain inoculation. Biomass was negatively correlated with DMC values because of the high water content of shoots. Growth (length, biomass, total biomass, and DMC) and physiological parameters (GPOX, chlorophyll) were not strongly correlated.

Growth parameters (length, biomass, total biomass, and DMC) and accumulation were strongly negatively correlated. In the case of high metal accumulation, the growth parameter values were lower, whereas the low accumulation values produced higher growth.

The GPOX activity of the root was positively correlated with root biomass, and both parameter values decreased. The root growth parameters and metal accumulation were strongly negatively correlated, as in the case of the shoot.

## 4. Discussion

### 4.1. Plant Growth Promoting Activity of the Bacterial Strains under Trace Metal Stress

Plant-growth-promoting bacteria (PGPR), which inhabit the rhizosphere and colonize the root surface, play beneficial roles that directly or indirectly affect plant health. In the present study, indigenous trace-metal-resistant bacteria were isolated, selected for their PGP traits, and tested for maize germination, growth and development under stress conditions. Two selected strains, *P. koreensis* BB2.A.1 and *S. liquefaciens* BB2.1.1, were able to solubilize phosphate; produce IAA, EPS, and siderophores; and maintain these characteristics under trace metal stress conditions. The ability of *Pseudomonas koreensis* to solubilize phosphate and produce IAA has been previously described for several strains [25,26]. Phosphate solubilization, IAA, siderophore, and EPS production by *Serratia liquefaciens* were reported by Samet et al. (2022) [27]. Siderophore and EPS production are important for preventing toxic metals from entering the cell and diminishing trace metal stress.

### 4.2. Effect of the Bacterial Treatment on Maize Seed Germination and Plant Growth under Trace Metal Stress

The effect of the two bacterial strains on the germination process was evaluated in vitro, and an increase in germination indices was observed. Inoculation with the *S. liquefaciens* BB2.1.1 bacterial strain decreased the growth parameters in the presence of metal treatments, whereas *P. koreensis* BB2.A.1 enhanced the growth parameters. Zarei et al. (2019) observed that the inoculation with the *Pseudomonas fluorescens* bacterial strain increased the growth of the sweet corn (*Zea mays* L. var saccharata) [28]. Moreira et al. (2016) showed that the metal-resistant *Pseudomonas fluorescens* S3X bacterial strain increases maize seedling growth when exposed to Cd and Zn [29]. Koo and Cho (2009) have shown that *Serratia plymuthica* SY5 isolated from soils contaminated with trace metals increased the growth of maize roots through the production of IAA [30]. Kurze et al. (2001) determined that the inoculation of strawberry plants with *Serratia plymuthica* bacteria increased the yield by 60% [31].

Based on our results, both bacterial strains decreased the shoot and root weights of maize plants, while the literature describes increasing trends. Shameer and Prasad (2018) observed in greenhouse experiments that inoculation with *Serratia plymuthica* increased the weight of cucumber plants by 29% [32]. The model PGPR strain *Serratia plymuthica* MBSA-MJ1 has the capacity to increase plant growth and plant tissue nutrient concentrations. Treatment with *S. plymuthica* MBSA-MJ1 influenced plant growth parameters in *Petunia hybrida* in Nordstedt and Jones (2021) [33]. Two *S. liquefaciens* strains increased potato plant growth, yield, and tuber quality [27].

In the case of the total biomass *P. koreensis* BB2.A.1 decreased the values in the presence of essential metal, while the toxic trace metal treatment increased the total biomass. The *Serratia liquefaciens* BB2.1.1 bacterial strain slightly decreased the total biomass. Moreira et al. (2016) observed that the *Pseudomonas fluorescens* bacterial strain inoculation significantly increased (18%) the maize biomass in the presence of Cd and Zn, and when the inoculum was doubled, the increase in biomass was 7% [29].

The shoot and the root DMC values were increased by the *P. koreensis* BB2.A.1 bacterial strain in the case of zinc treatment, whereas toxic trace metals decreased DMC. The *Serratia liquefaciens* BB2.1.1 bacterial strain inoculation showed the opposite results. Asilian et al. (2019) did not observe any significant differences in the maize shoot and the root dry weight in the presence of Cd treatment [34]. Wu et al. (2020) observed that the use of the *Pseudomonas fluorescens* bacterial strain increased the shoot and the root dry weight in the presence of cadmium treatment, compared to the control [35]. The dry weight and fruit yield of cucumber (*Cucumis sativus*) were increased by *Serratia plymuthica* based on Egamberdieva and Lugtemberg (2013) [36,37].

### 4.3. Trace Metal Accumulation in Maize Plants

The two bacterial strains used (BB2.A.1 and BB2.1.1) decreased the accumulation of Cd in the shoot in the presence of the combined treatment. Similar results were observed by Moreira et al. (2016) because Cd accumulation was decreased in maize roots when plants were inoculated with *Pseudomonas fluorescens* at a low inoculum size [29]. When the inoculum size was doubled, accumulation was enhanced in the roots. In the case of shoots, they found that inoculation with the *Pseudomonas fluorescens* bacterial strain promoted the accumulation of cadmium in both inoculum sizes [29]. Chiboub et al. (2020) demonstrated that the inoculation with a consortium (*Pseudomonas* sp., *Pseudomonas fluorescens*, and two *R. sullae*) enhanced Cd accumulation in the *Sulla coronaria* Bikra 21 root (57%) more than in the shoot (15%) [38]. According to Wu et al. (2020), increased dry biomass contributes more to the improvement of Cd accumulation when inoculated with *Pseudomonas fluorescens*, compared to increased Cd concentration [35]. Asilian et al. (2019) reported that *Pseudomonas fluorescens* was effective in increasing Cd concentration in the maize shoot [34]. Tan et al. (2020) observed Zn and Cd accumulation and translocation in rice plants in the presence of a combined treatment [15]. The amount of accumulated zinc was higher than that of cadmium. The effects of the two studied bacterial strains, *Pseudomonas koreensis* and *Serratia liquefaciens*, on maize growth under trace metal stress have not been previously reported.

The *Pseudomonas koreensis* BB2.A.1 and *Serratia liquefaciens* BB2.1.1 bacterial strains stimulated zinc accumulation in the shoots and roots with zinc treatment, whereas a decrease in Zn accumulation was observed in the presence of combined treatment, most probably caused by Cd competition. Tan et al. (2020) highlighted that in rice, Cd hijacks essential micronutrient pathways through putative metal cation transporters to accumulate in plants [15].

Moreira et al. (2016) showed that in the case of Zn accumulation, the inoculum size had no significant effect on maize plants. Nevertheless, the *Pseudomonas fluorescens* bacterial strain decreased zinc accumulation in the roots regardless of the size of the inoculum, and a general increase in zinc accumulation was detected in the shoot. In the maize shoot, the *P. fluorescens* bacterial strain was able to enhance zinc accumulation by 67% and 56% when the inoculum was doubled, compared to the uninoculated samples [29]. Fuloria et al. (2009) observed increased root and shoot uptake of Zn in *B. juncea* by 3.05 and 2.69 times, when soil was inoculated with the *P. fluorescens* bacterial strain [39]. Neither of the studied strains, *Pseudomonas koreensis* nor *Serratia liquefaciens*, have been studied in previous research related to zinc accumulation in plants.

The accumulated Cd in the root cannot be transported to the shoot in the case of combined treatment in the presence of both bacterial strains; however, the TF values were increased by the Cd treatment. The translocation of zinc was observed to a large extent with both bacterial strain inoculations. Similar results were reported by Asilian et al. (2019), where the translocation factor of cadmium was reduced when inoculated with the *Pseudomonas fluorescens* bacterial strain [34]. Fuloria et al. (2009) observed that TF values < 1 indicate low metal translocation to the shoot, which can be attributed to the strong binding capacity of metals to root cells and/or compartmentalization into the vacuoles, restricting the movement of metals from the roots to the shoot. Their research on *B. juncea* and with the *Pseudomonas fluorescens* bacterial strain inoculation resulted in the order Zn > Cd > Cu in TF, which may be due to the fact that cadmium and zinc are more mobile metals and were hence bound less strongly by the soil matrix as compared to copper [39].

### 4.4. Effect of Bacterial Treatment on Maize Physiology under Trace Metal Stress

Our results did not show statistically significant differences in the total chlorophyll content of the maize plants, whereas inoculation with the *P. fluorescens* bacterial strain resulted in 1.29 times higher chlorophyll content in the cadmium-treated samples compared to the control, based on Wu et al. (2020) [35]. The *Serratia plymuthica* bacterial strain was not used in similar plant experiments, and there is no literature on its effect on chlorophyll content. The Chla/b ratio was higher in several cases because of the reduced Chlb content compared with that of Chla. A higher Chla/b ratio provides plants with a greater ability to tolerate stress [40].

The *Pseudomonas koreensis* BB2.A.1 bacterial strain increased shoot GPOX activity in all treatments. Plants inoculated with this strain also had a higher Chla/b ratio in the leaves. Both physiological indicators are related to improved stress tolerance, which was induced by inoculation with the *Pseudomonas koreensis* BB2.A.1 bacterial strain. Root GPOX activity was decreased by the same bacterial strain in the presence of zinc. The *Serratia liquefaciens* BB2.1.1 bacterial strain increased GPOX activity in the shoot and root in the presence of zinc, while cadmium treatment decreased the activity. The bacterial inoculation induced the level of protective enzyme, that is beneficial in the abiotic stress condition. Chiboub et al. (2020) related that inoculation with a consortium (*Pseudomonas* sp., *Pseudomonas fluorescens*, and two *R. sullae*) in the presence of Cd treatment inhibited GPOX activity by 21%, and the exposure to double Cd concentration enhanced enzyme activity by 240% [38]. There are no data in the literature regarding the effect of the *Pseudomonas koreensis* and *Serratia liquefaciens* bacterial strains on GPOX enzyme activity.

## 5. Conclusions

The two trace-metal-resistant PGP bacterial strains used in the plant experiments showed positive effects on maize germination according to most of the germination indices and several growth parameters under trace metal stress conditions. *Pseudomonas koreensis* BB2.A.1 was more efficient in promoting plant growth; in the Cd-containing treatments, the biomass of plants was higher than that of the control or other treatments. Concomitantly, in the case of Cd-containing treatments, the antioxidant activity and Chla/b ratio observed in the plants was also increased by bacterial inoculation with *Pseudomonas koreensis* BB2.A.1, indicating a stress tolerance induction. The chlorophyll content was positively affected by *Serratia liquefaciens* BB2.1.1 bacterial strain inoculation in all treatments. In terms of the accumulation, a reduced accumulation of the toxic metal (Cd) and a stimulated accumulation of the trace element (Zn) was observed in plants inoculated with *Serratia liquefaciens* BB2.1.1 bacterial strain. The two studied bacterial strains were found to be less efficient in diminishing the negative effect of trace metal treatment on the length of the shoots and roots of maize plant.

Both bacterial strains efficiently colonized the rhizosphere and have been proven to have beneficial effects on trace-metal-stressed maize plants in different ways, having potential in sustainable agricultural practice. Due to the fact that they complement each other, their synergisms and further possibilities in using them as bioinoculants in a consortium remain to be exploited. The effectiveness of bacterial isolates should also be tested under field conditions. The optimization of bacterial isolates for proper fermentation and formulation processes is required to achieve success in the agricultural field. The use of such bioinoculants is important not only for sustainable agricultural production, but also for food security issues.

## Figures and Tables

**Table 1:** Beneficial traits of the isolated bacterial strains (means are followed by standard deviance (n = 3); different letters represent significant differences among the bacterial strains according to Tukey’s test, *p* < 0.05).

Su	PGPR Traits				Motility	
	Siderophore (Halo mm)	Inorganic Phosphorus (Halo mm)	IAA Production (mg/mL)	EPS Production (OD_570_)	Swimming (mm)	Swarming (mm)
BB1.A.1	0 ± 0	0 ± 0	0.04 ± 0.01 a	0.12 ± 0.00 a	7.43 ± 1.37	8.33 ± 1.15
BB1.A.2	13.06 ± 0.53 a	8.41 ± 0.78 a	0.13 ± 0.00 b	0 ± 0	6.50 ± 0.50	8.50 ± 0.87
BB1.A.3	6.23 ± 0.85 b	11.23 ± 0.56 b	0.15 ± 0.00 b	0.30 ± 0.01 b	9.00 ± 0	8.17 ± 1.04
BB1.1.2	7.34 ± 2.79 b	2.13 ± 0.58 c	0.08 ± 0.00 a	0.09 ± 0.00 a	8.00 ± 0	4.07 ± 2.00
BB1.1.3	13.15 ± 0.02 a	7.22 ± 0.89 a	0.56 ± 0.02 c	0.10 ± 0.03 a	5.33 ± 0.58	8.33 ± 0.58
BB2.A.1	11.40 ± 0.66 a	8.23 ± 0.42 a	0.60 ± 0.02 d	0.08 ± 0.03 a	7.67 ± 0.58	9.00 ± 0
BB2.1.1	11.01 ± 0.59 a	9.78 ± 0.50 ab	0.41 ± 0.00 e	0.19 ± 0.01 b	9.00 ± 0	6.5 ± 0.87
BB2.2.1	11.40 ±0.82 a	7.10 ± 0.52 a	0.64 ± 0.03 d	0.09 ± 0.03 a	8.00 ± 1.73	9.00 ± 0
BB2.2.2	8.38 ± 0.21 b	0 ± 0	0.07 ± 0.00 a	0.02 ± 0.02 c	7.20 ± 0.20	2.53 ± 0.75
BB2.3.1	8.00 ± 0.74 b	0 ± 0	0.06 ± 0.00 a	0.14 ± 0.10 a	8.83 ± 0.29	5.03 ± 0.90
BB3.A.1	0 ± 0	0 ± 0	0.36 ± 0.00 e	0.24 ± 0.01 b	1.97 ± 0.35	5.10 ± 3.80

**Table 2:** Plant growth promoting characteristics of the selected strains under trace metal stress.

Bacterial Strains	Trace Metal Treatments	PGPR Properties				
		Siderophore (Halo mm)	Organic Phosphorus (Halo mm)	Inorganic Phosphorus (Halo mm)	IAA Production (mg/mL)	EPS Production (OD570)
BB2.A.1	Control	11.40 ± 0.66 a	0 ± 0	8.23 ± 0.42 a	0.60 ± 0.02 a	0.08 ± 0.03 a
	1 mM Zn	16.53 ± 0.50 b	8.18 ± 0.39 a	6.06 ± 0.83 b	0.46 ± 0.02 b	0.16 ± 0.02 ab
	0.5 mM Cd	3.26 ± 0.09 c	6.95 ± 0.31 a	6.19 ± 0.54 b	0.48 ± 0.02 b	0 ± 0
	0.1 mM Cd + 0.5 mM Zn	21.66 ± 1.88 d	12.60 ± 2.00 b	7.39 ± 0.72 a	0.50 ± 0.02 b	0 ± 0
BB2.1.1	Control	11.01 ± 0.59 a	0 ± 0	9.78 ± 0.50 a	0.41 ± 0.004 c	0.19 ± 0.01 bc
	1 mM Zn	18.45 ± 1.24 d	10.72 ± 0.73 a	9.61 ± 0.63 a	0.40 ± 0.005 c	0.26 ± 0.01 c
	0.5 mM Cd	5.89 ± 0.87 c	13.07 ± 0.22 b	7.64 ± 0.64 a	0.35 ± 0.006 cd	0 ± 0
	0.1 mM Cd + 0.5 mM Zn	21.14 ± 0.56 d	16.16 ± 0.73 c	9.65 ± 0.29 a	0.34 ± 0.006 d	0.15 ± 0.04 ab

Means and SD values followed by different letters represent significant differences among the treatments according to Tukey’s test, *p* < 0.05 (n = 3).

**Table 3:** The impact of the inoculation with the selected bacterial strains on maize seed germination indices (n = 8).

	Germination Rate, G (%)	Mean Germination Time, MGT (day)	Coefficient of Velocity of Germination, CVG (%day^−1^)	Mean Germination Rate, MGR (day^−1^)	Germination Rate Index, GRI (%day^−1^)	Germination Index, GI	Timson Germination Index, TGI (%)
Control	48.78 ± 23.68	1.96 ± 0.05	21.76 ± 15.58	0.21 ± 0.15	47.03 ± 22.43	91.00 ± 43.22	6.09 ± 2.96
BB2.A.1	51.25 ± 24.71	1.98 ± 0.02	24.43 ± 19.20	0.24 ± 0.19	50.57 ± 22.70	97.75 ± 44.15	6.40 ± 3.08
BB2.1.1	61.25± 21.46	1.91 ± 0.08	29.97 ± 16.68	0.39 ± 0.16	55.36± 22.07	107.75 ± 42.49	7.65 ± 2.68

**Table 4:** The average and standard deviation values of the shoot and root length.

Treatments	The Length of the Shoot (cm)		
	CONTROL	BB2.A.1	BB2.1.1
Control	48.2 ± 4.71 ab	43.88 ± 8.58 b	44.46 ± 2.94 ab
1 mM Zn	46.83 ± 2.57 ab	46.39 ± 4.02 ab	47.95 ± 6.63 b
0.5 mM Cd	48.89 ± 4.73 ab	46.53 ± 3.18 ab	43.26 ± 8.06 ab
0.5 mM Zn + 0.1 mM Cd	52.08 ± 2.74 a	48.43 ± 3.38 ab	44.46 ± 5.41 ab
**Treatments**	**The length of the root (cm)**		
	**CONTROL**	**BB2.A.1**	**BB2.1.1**
Control	19.82 ± 3.26 adg	20.03 ± 2.75 acd	16.1 ± 2.50 cdgi
1 mM Zn	21.34 ± 3.36 abdgh	13.83 ± 2.58 i	11.71 ± 2.07 fgij
0.5 mM Cd	23.07 ± 6.60 adh	15.55 ± 2.21 cdghi	14.43 ± 3.03 fgi
0.5 mM Zn + 0.1 mM Cd	16.87 ± 2.06 cdegi	16.14 ± 3.05 cdfgi	11.44 ± 1.00 fgi

Different letters represent significant differences among the treatments according to Tukey’s test, *p* < 0.05.

**Table 5:** The average and standard deviation values of shoot and root biomass.

Treatments	The Biomass of the Shoot (g)		
	CONTROL	BB2.A.1	BB2.1.1
Control	1.44 ± 0.23 a	1.08 ± 0.35 ah	1.32 ± 0.25 ad
1 mM Zn	1.46 ± 0.24 a	0.86 ± 0.32 befgh	0.94 ± 0.31 bcdefgh
0.5 mM Cd	1.21 ± 0.36 af	1.25 ± 0.23 aef	1.00 ± 0.36 bcdefgh
0.5 mM Zn + 0.1 mM Cd	1.33 ± 0.15 ac	1.46 ± 0.22 a	1.11 ± 0.21 ag
**Treatments**	**The biomass of the root (g)**		
	**CONTROL**	**BB2.A.1**	**BB2.1.1**
Control	0.54 ± 0.06 ag	0.54 ± 0.05 af	0.45 ± 0.08 bcdefg
1 mM Zn	0.58 ± 0.07 ac	0.44 ± 0.15 bdefg	0.58 ± 0.07 a
0.5 mM Cd	0.56 ± 0.10 ae	0.61 ± 0.07 a	0.4 ± 0.08 b
0.5 mM Zn + 0.1 mM Cd	0.36 ± 0.02 b	0.56 ± 0.08 ad	0.43 ± 0.05 bfg

Different letters represent significant differences among the treatments according to Tukey’s test, *p* < 0.05.

## Data Availability

The raw data supporting the conclusions of this article will be made available by the authors on request.
